# Yiqi Fumai Lyophilized Injection for Improving Exercise Tolerance in Chronic Heart Failure: Protocol for a Prospective Cohort Study

**DOI:** 10.2196/91051

**Published:** 2026-07-07

**Authors:** Yiming Zuo, Yunfeng Jia, Lu Fan, Yayi Liu, Yuxiao Cao, Miaomiao Wei, Lei Wei, Xiaoling Wang, Xuezheng Liu, Shichao Lv

**Affiliations:** 1 Department of Geriatrics, First Teaching Hospital of Tianjin University of Traditional Chinese Medicine, National Clinical Research Center for Chinese Medicine, No 88 Changling Road, Tianjin, 300381, China, 86 13920127740; 2 Department of Geriatrics, Qian'an Hospital of Traditional Chinese Medicine, Tangshan, China

**Keywords:** Yiqi Fumai lyophilized injection, chronic heart failure, exercise tolerance, cohort study, cardiac rehabilitation

## Abstract

**Background:**

High mortality and prevalence rates are hallmarks of chronic heart failure (CHF). Patients frequently have a much lower quality of life as a result of diminished exercise tolerance. Chinese guidelines have recommended Yiqi Fumai lyophilized injection (YQFM) for the treatment of heart failure, although there is currently inadequate evidence to support its effectiveness in increasing exercise tolerance in these patients.

**Objective:**

The purpose of this cohort study is to examine the relationship between the improvement of exercise tolerance in patients with CHF and the addition of YQFM to guideline-directed medical therapy.

**Methods:**

In total, 216 hospitalized patients with CHF with New York Heart Association (NYHA) functional classes II-IV were to be enrolled in the prospective, observational cohort trial design. The participants were divided into a YQFM group (exposed group: n=144) and a non-YQFM group (nonexposed group: n=72) at a 2:1 ratio based on real clinical medication and patient preference. Standard guideline-directed medical therapy was administered to both groups; however, the YQFM group also got a 10-day YQFM exposure. The change in metabolic equivalents measured by the Veterans Specific Activity Questionnaire was the main outcome. The 6-minute walk distance, Kansas City Cardiomyopathy Questionnaire score, NYHA functional class, N-terminal pro-B-type natriuretic peptide levels, and echocardiographic parameters were among the secondary objectives. Traditional Chinese medicine syndrome scores and the frequency of hard clinical occurrences were the exploratory objectives. A linear mixed-effects model was used to examine repeated measurement data, and propensity score weighting was used to account for baseline confounding variables.

**Results:**

The first patient was registered in June 2024, and all 216 patients had been recruited and followed up with by December 2025. Data cleaning and statistical analysis began in January 2026, with final results scheduled to be published in the autumn of 2026.

**Conclusions:**

This study, using a prospective cohort design, intends to offer high-quality, real-world evidence for the use of YQFM in cardiac rehabilitation for heart failure. This will aid in the optimization of preventative and treatment strategies for CHF that combine traditional Chinese and Western medicine, giving an objective basis for enhancing patients’ long-term quality of life.

## Introduction

Due to its consistently high hospitalization and mortality rates, heart failure (HF), a severe manifestation or the final stage of cardiovascular disease, represents a significant global disease burden [1]. The 2022 American Heart Association/American College of Cardiology/Heart Failure Society of America Guideline for the Management of Heart Failure recommends upgrading the medical therapy for HF from the conventional “golden triangle” to a “new quadruple therapy,” which emphasizes the principles of individualized and comprehensive treatment, due to the ongoing accumulation of clinical evidence for medications like angiotensin receptor-neprilysin inhibitors and sodium-glucose cotransporter-2 inhibitors in improving the long-term prognosis of HF [2]. Nonetheless, there are several variables, targets, and pathologic pathways involved in the intricate pathophysiological mechanisms of HF. Patients are still at risk for HF deterioration and death even with “quadruple therapy,” which addresses metabolic and neuroendocrine pathways at the same time [3]. Vericiguat, a soluble guanylate cyclase stimulator, can successfully lower cardiovascular mortality and HF hospitalization rates by working on intracellular signaling pathways in addition to standard therapy [4]. As a result, vericiguat has been progressively included in the recommendations of subsequent HF guidelines. When combined with the “new quadruple therapy,” it creates the new framework for guideline-directed medical therapy (GDMT), a multimodal treatment approach that represents 5 important pharmacological pillars.

Even though medical theories and therapies for chronic heart failure (CHF) are constantly evolving, many problems still exist in certain areas, such as diuretic resistance, aldosterone escape, and a markedly lower quality of life with no viable therapy alternatives [5,6]. One of the most prevalent signs of HF is decreased exercise tolerance, which is a major factor in the loss of quality of life among patients with HF [7]. Therapy with exercise for patients with CHF is still relatively new in China and does not yet have clear, accepted recommendations. Even serious adverse outcomes like sudden cardiac death might be brought on by inappropriate exercise training. The 2018 Chinese Guidelines for the Diagnosis and Treatment of Heart Failure recommendations for cardiac rehabilitation prescriptions state that drugs such as trimetazidine and β-blockers can increase exercise tolerance [8]. However, individuals with bradycardia and bronchial asthma should not take β-blockers, and trimetazidine may produce neuromuscular symptoms, which significantly restricts its clinical application [9]. In patients with HF, traditional Chinese medicine (TCM) has shown therapeutic promise in reducing clinical symptoms, increasing exercise tolerance, and improving quality of life [10]. However, there are few clinical trials using the improvement of exercise tolerance as the major objective, and prior research on HF has mostly concentrated on long-term outcomes (such as readmission or mortality rates). Yiqi Fumai lyophilized injection (YQFM) is a lyophilized powder formulation developed using modern technologies, composed of Red Ginseng (Radix Ginseng Rubra), Maidong (Radix Ophiopogonis), and Wuweizi (Fructus Schisandrae). Approved by the National Medical Products Administration in 2007, YQFM is indicated for the treatment of cardiovascular diseases, including chronic cardiac insufficiency secondary to coronary heart disease and stable angina pectoris. Numerous clinical recommendations and expert consensuses have approved it because of its demonstrated safety and efficacy.

Previously, with the support of the East China Institute of Cardiovascular Health, our team conducted a single-center, prospective, randomized study demonstrating the efficacy of YQFM intervention in improving exercise tolerance among patients with CHF [11]. However, prior research has suggested that there can be differences between actual clinical practice and the outcomes of randomized controlled trials (RCTs). RCTs’ stringent inclusion and exclusion criteria frequently result in the underrepresentation of specific patient categories (such as those with complicated comorbidities), which reduces the applicability of experimental medications in actual clinical settings [12,13]. Thus, this study takes a patient-centered and guideline-directed research strategy, expanding on our earlier work. We concentrate on patients with CHF using a population-based cohort research design, with YQFM serving as the exposure variable and exercise tolerance as the main emphasis. This study intends to address the shortcomings of RCTs, offer a practical assessment applicable to a wider range of patients in routine clinical practice, and clarify the clinical impact of YQFM in improving exercise tolerance among patients with CHF, thereby advancing the prevention and treatment of HF, by using observational data from real-world research.

## Methods

### Study Design

A prospective, observational cohort study design was used in this investigation. Patients with a verified diagnosis of CHF who all got standard GDMT made up the study population. Real-world clinical prescription decisions and actual medication use at the time of enrollment were used to determine the exposure status. In the meantime, the investigators did not interfere with the clinical prescription decisions, and the patients’ treatment preferences and informed consent were fully respected. Based on whether or not they received YQFM treatment in addition to regular therapy throughout their hospital stay, the patients were divided into the YQFM group and the non-YQFM group in accordance with the principle of respecting shared decision-making between doctors and patients. Patients were instructed to minimize concurrent use of additional TCM injections, proprietary Chinese medications, or TCM decoctions during the 10-day exposure period and subsequent follow-ups. If such medications were strictly necessary due to concurrent acute illnesses (eg, severe infections), the investigators recorded the generic drug names, dosages, and start or stop dates in detail. These were treated as time-varying covariates for adjustment or exclusion during the statistical analysis phase.

**Figure 1.**
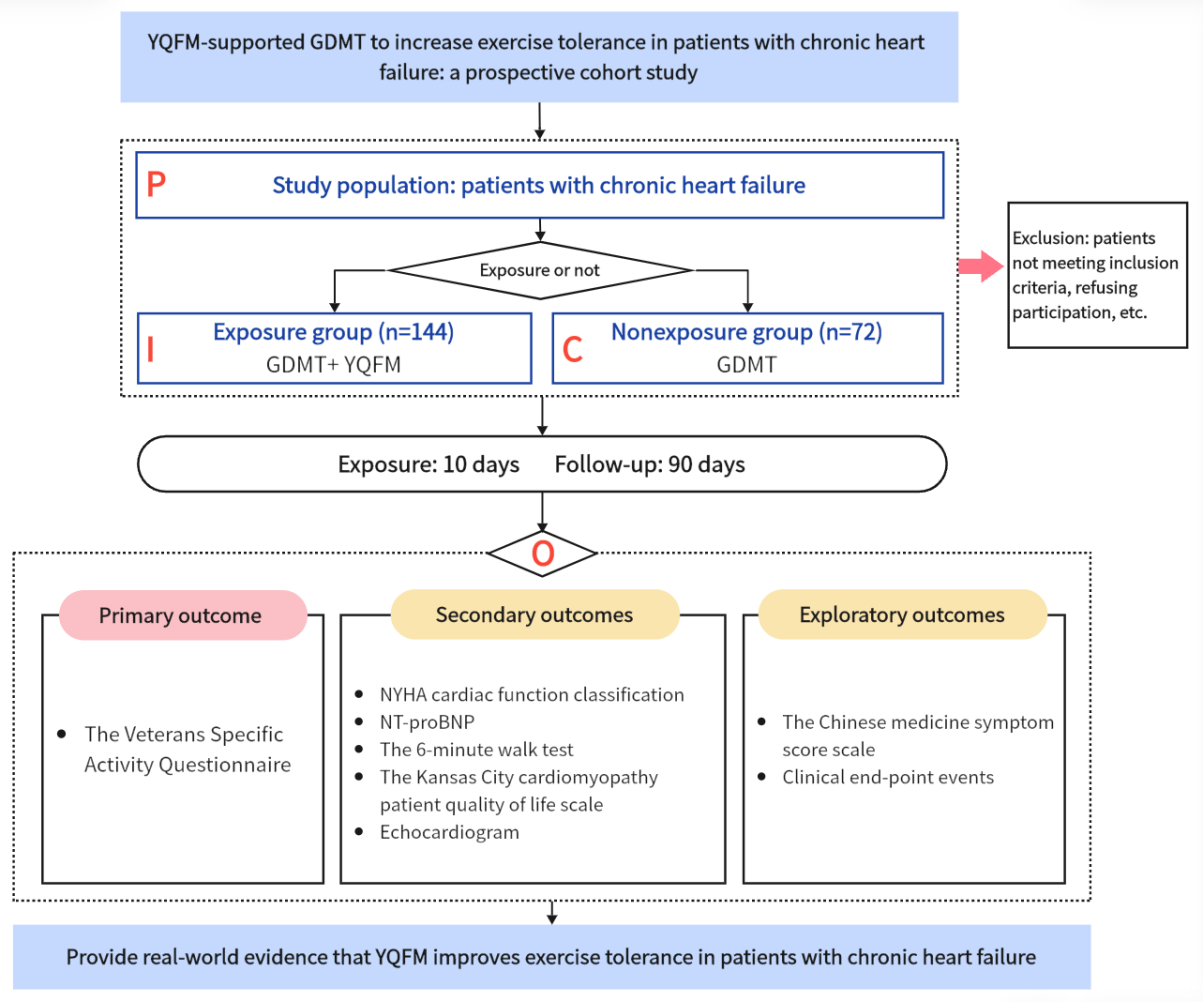
Technical roadmap. The flowchart illustrates the study framework based on the participants, intervention, comparison, outcomes, and study design principle. Patients with chronic heart failure are divided into 2 cohorts: the YQFM group (GDMT+YQFM) and the non-YQFM group (GDMT alone) according to real-world clinical practice and patient preference. The study design comprises a 10-day exposure period followed by a 90-day follow-up phase (concluding at day 100) to evaluate primary, secondary, and exploratory outcomes. GDMT: guideline-directed medical therapy; NT-proBNP: N-terminal pro-B-type natriuretic peptide; NYHA: New York Heart Association; YQFM: Yiqi Fumai lyophilized injection.

The “target trial emulation” concept was used in this study’s design to reduce selection bias and confounding by indication brought on by nonrandomized allocation. To guarantee homogeneity between the exposed and nonexposed groups at the beginning of the follow-up, the study rigorously aligned the baseline “time zero” (ie, the precise moment when patients met the inclusion and exclusion criteria and the treatment plan was finalized). To provide solid data support for later statistical adjustments, the study also thoroughly gathered important covariates that are strongly linked to the main outcome (exercise tolerance) and treatment propensity (eg, age, NYHA functional class, baseline Veterans Specific Activity Questionnaire [VSAQ] score, attainment of GDMT targets, and comorbidities). The overall framework and technical roadmap of this study are illustrated in Figure 1.

### Study Population

#### Diagnostic Criteria

##### Diagnostic Criteria for CHF

The diagnosis of CHF was based on the Chinese guidelines for the diagnosis and treatment of heart failure 2024 [14]. Patients must present with symptoms and/or signs of HF (eg, dyspnea, fatigue, anorexia, and bilateral lower extremity edema), accompanied by an elevated B-type natriuretic peptide (BNP)>35 ng/L and/or N-terminal pro-B-type natriuretic peptide (NT-proBNP)>125 ng/L, as well as objective evidence of structural and/or functional cardiac abnormalities on echocardiography. Concurrently, HF phenotypes were standardized, classified, and defined based on the baseline echocardiographic left ventricular ejection fraction (LVEF). Patients were categorized into 3 groups: HF with reduced ejection fraction (LVEF≤40%), HF with mildly reduced ejection fraction (LVEF 41%‐49%), and heart failure with preserved ejection fraction (LVEF≥50%). For patients with HF with mildly reduced ejection fraction and HF with preserved ejection fraction, concurrent objective criteria of structural and functional abnormalities on echocardiography, such as left ventricular hypertrophy, left atrial enlargement, or diastolic dysfunction, were required.

##### Diagnostic Criteria for Qi and Yin Deficiency Syndrome

The diagnosis was based on the Technical Guiding Principles for Clinical Research of New Traditional Chinese Medicines in the Treatment of Chronic Heart Failure.

- Primary symptoms: Shortness of breath or panting, fatigue or asthenia, and palpitations.
- Secondary symptoms: Thirst or dry throat; spontaneous sweating or night sweats; dysphoria in the chest, palms, and soles (5-palm heat); and dark purplish complexion, mouth, and lips.
- Tongue and pulse manifestations: A thin tongue body; a dark tongue texture (or with petechiae, ecchymosis, or tortuous and dark purplish sublingual collateral vessels); scanty, absent, or fissured tongue coating; and a thready and weak pulse.

Confirmation rule: In addition to the typical tongue and pulse presentations, a diagnosis must have at least 2 primary symptoms and 2 secondary symptoms.

### Inclusion and Exclusion Criteria

To ensure the homogeneity of the study population and the safety of the patients, the specific inclusion and exclusion criteria are detailed in Textbox 1.

Textbox 1. **Inclusion and exclusion criteria.**

**Inclusion criteria**

- Meet the diagnostic criteria for chronic heart failure with Qi and Yin deficiency syndrome.
- Aged 18 to 75 years, both sexes.
- New York Heart Association functional class II-IV.
- Patients who willingly sign the informed consent form after fully understanding the study’s protocols.

**Exclusion criteria**

- Concurrent acute or critical cardiovascular diseases, such as acute coronary syndrome (within 30 days), cardiogenic shock, acute myocarditis, uncontrolled hypertension (systolic blood pressure≥180 mm Hg and/or diastolic blood pressure≥110 mm Hg), uncontrolled malignant arrhythmias, hypertrophic obstructive cardiomyopathy, severe valvular heart disease requiring surgical intervention, or pulmonary embolism.
- Concurrent severe hepatic or renal dysfunction or known hypersensitivity to the study medications.
- Presence of malignant tumors, severe mental disorders, or other conditions that may prevent the completion of follow-up.
- Pregnant or lactating women.

### Sample Size

The longitudinal change in metabolic equivalents (METs) over the course of the follow-up period was the main end point of this study, and the key reference for the sample size calculation was the between-group difference in METs at 90 days after exposure. Based on previously published clinical trial data concerning similar TCM adjunctive therapies for CHF, combined with real-world clinical expectations, the estimated mean of METs was approximately 4.78 (SD 0.91) in the exposed group (YQFM group) and 4.34 (SD 1.08) in the nonexposed group (non-YQFM group) [15]. The estimated between-group difference of 0.44 METs met the threshold for a clinically meaningful improvement. The sample size allocation ratio between the exposed and nonexposed groups was set at 2:1. The 2-sided significance level (*α*) was set at 0.05 (corresponding to *Z*_1−*α*/2_=1.96), and the statistical power (1−*β*) was set at 0.80 (corresponding to *Z*_1−*β*_=0.84). For the unequal allocation design, the following estimation formula for comparing the means of 2 independent samples was used:


$$n_{c}=\frac{(Z_{1-\alpha /2}+Z_{1-\beta }{)}^{2}\times \left(\frac{S_{e}^{2}}{k}+S_{c}^{2}\right)}{(\mu_{e}-\mu_{c}{)}^{2}}$$

The required base sample size to finish the follow-up was determined to be 64 patients in the nonexposed group and 128 patients in the exposed group, for a total of 192 patients, by substituting these parameters into the formula. Accounting for an estimated 10% attrition and loss to follow-up rate during the 90-day follow-up period, the final planned enrollment was determined to be 72 patients in the nonexposed group and 144 patients in the exposed group, yielding a total of 216 required patients. The statistical power (1−*β*) was initially prespecified at the standard clinical trial threshold of 0.80 (80%) for a conservative, single-time-point sample size estimation (90 days after exposure). The planned sample size of 216 patients was calculated to meet this 80% baseline. However, in our actual statistical plan, the primary analysis uses a linear mixed-effects model (LMM) incorporating all 4 repeated measurements. In longitudinal designs, LMM accounts for intrasubject correlation, which significantly reduces the residual error variance compared to a single-point cross-sectional analysis. Because of this increased statistical efficiency, the effective sample size provided by the longitudinal data is substantially larger. Therefore, the actual statistical power for detecting the treatment-by-time interaction with these 216 patients will exceed the initially preset 80% threshold.

### Interventions

From the time of hospital admission to the conclusion of the follow-up period, all patients received standardized GDMT in compliance with the 2022 American Heart Association/American College of Cardiology/Heart Failure Society of America Guideline for the Management of Heart Failure [2]. The use, titration algorithms, and actual target dose achievement rates of key GDMT medication classes (angiotensin receptor-neprilysin inhibitor or angiotensin-converting enzyme inhibitor or angiotensin II receptor blocker, β-blockers, mineralocorticoid receptor antagonists, and sodium-glucose cotransporter-2 inhibitors) were thoroughly reported in this study, which provided a strict operational definition for GDMT treatment. Patients who did not meet the guideline-recommended target dosages or were unable to use a specific core medication class had their reasons for nonattainment meticulously documented (eg, symptomatic hypotension, bradycardia, deteriorating renal function, hyperkalemia, or subjective patient rejection). Based on whether they received YQFM treatment during hospitalization, and under the premise of respecting patient autonomy, the patients were allocated into the non-YQFM group and the YQFM group. The exposure period for the Yiqi Fumai injection was 10 days.

- Non-YQFM group: Received only the standardized and operationally tracked GDMT described earlier.
- YQFM group: Received YQFM in addition to the treatment of the non-YQFM group (Tasly Pharmaceutical Group Co, Ltd, National Medical Products Administration Approval No: Z20060463; 5.2 g once daily, dissolved in 250 mL of 5% glucose or 0.9% normal saline).

### Study Schedule

#### Overview

**Figure 2.**
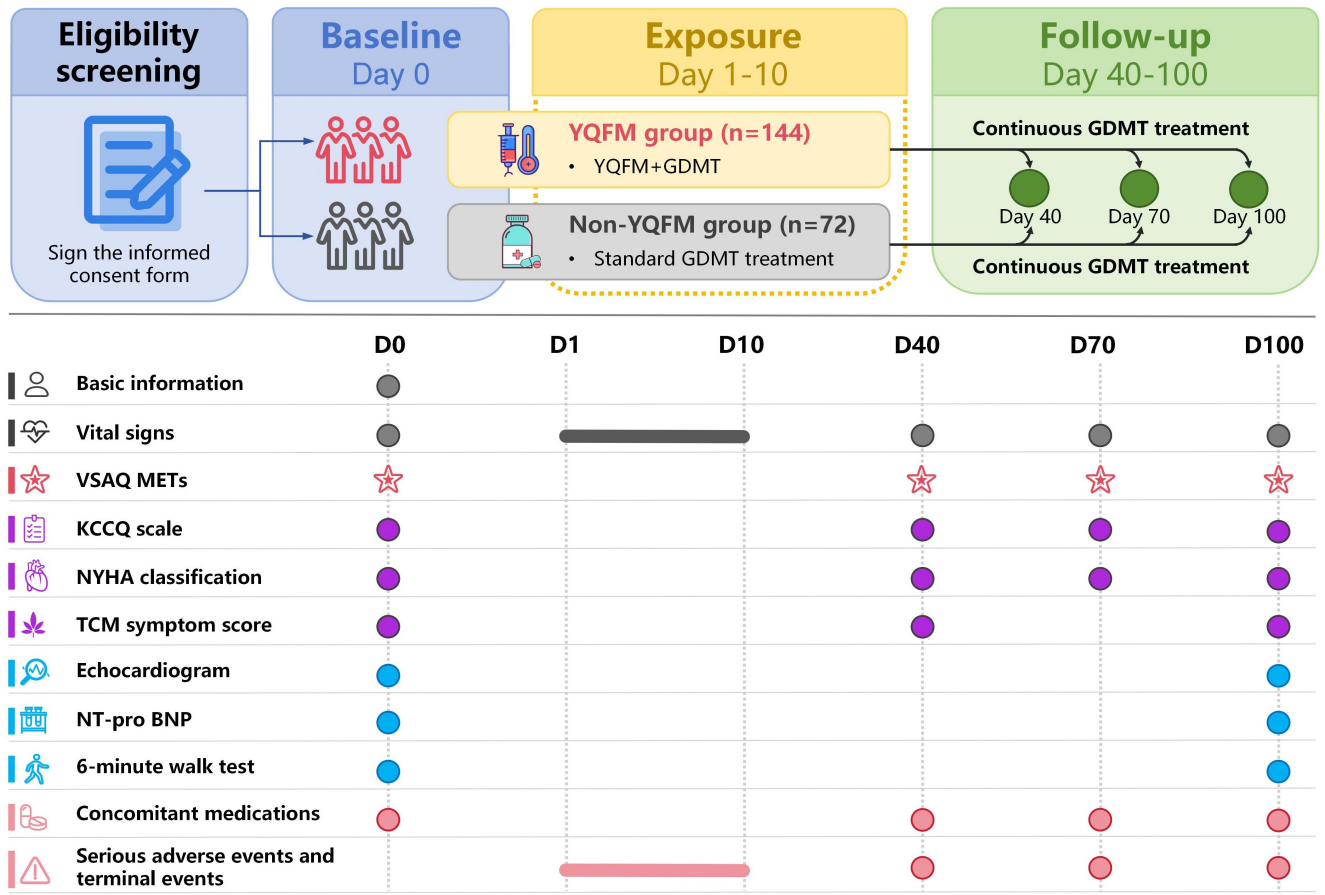
Study assessment schedule and timeline. This visual timeline depicts the comprehensive clinical assessment plan at baseline (day 0), during the hospital exposure period (days 1-10), and at the follow-up intervals (days 40, 70, and 100). Vital signs and potential adverse reactions are monitored daily during the 10-day intervention. Key functional and quality-of-life scales (VSAQ, KCCQ, and NYHA classification) are reassessed at each follow-up visit, while objective laboratory and imaging parameters (echocardiogram, NT-proBNP, and 6-minute walk test) are repeated at the final study end point to evaluate long-term structural and functional recovery. GDMT: guideline-directed medical therapy; KCCQ: Kansas City Cardiomyopathy Questionnaire; MET: metabolic equivalent; NT-proBNP: N-terminal pro-B-type natriuretic peptide; NYHA: New York Heart Association; TCM: traditional Chinese medicine; VSAQ: Veterans Specific Activity Questionnaire; YQFM: Yiqi Fumai lyophilized injection.

The baseline phase (day 0), the exposure period (days 1‐10), and the follow-up period (days 40, 70, and 100) were the prespecified time points at which the clinical data collection and follow-up evaluations in this study were carried out. The detailed study assessment schedule and follow-up timeline are illustrated in Figure 2.

#### Baseline (Day 0)

The patients signed the informed consent form on the day of enrollment. In addition to current concurrent medications, the researchers gathered baseline data on comorbidities, smoking and alcohol history, disease duration, and demographics. The NYHA functional class and baseline vital indicators (heart rate, blood pressure, and respiration rate) were noted. At the same time, objective ancillary tests (such as the 6-minute walk test [6MWT], echocardiography, and NT-proBNP levels) and subjective questionnaire assessments (such as the VSAQ, the Kansas City Cardiomyopathy Questionnaire [KCCQ], and TCM syndrome scores) were completed.

#### Exposure Period (Days 1-10)

During this period, patients’ vital signs were closely monitored and recorded daily. Concurrently, the occurrence of serious adverse events (SAEs) and clinical end-point events was tracked and documented throughout the entire process.

#### Follow-Up Period (Days 40, 70, and 100)

Patients’ vital signs, current concomitant drug status, VSAQ and KCCQ scores, and NYHA functional class were reviewed at each follow-up visit, with SAEs and end-point events monitored continuously. TCM syndrome scores were only reviewed at the early (day 40) and ultimate (day 100) follow-ups after exposure. At the study’s last follow-up visit (day 100), participants had a complete review that included echocardiography, NT-proBNP, and the 6MWT to objectively measure long-term improvements in heart structure, neurohormonal state, and exercise tolerance.

### Primary Outcome Measure

The exact primary end point is the longitudinal change from baseline in VSAQ-predicted METs over the 90-day follow-up period after exposure (specifically evaluated at day 40, day 70, and day 100 of the study). The VSAQ is a self-administered questionnaire comprising 13 routine activities ranked in progressive order of energy expenditure (1 to 13 METs). Patients identify the activity threshold that causes symptoms such as fatigue or shortness of breath. To accurately convert the VSAQ responses into objective METs, we will use the widely validated Myers regression equation incorporating the patient’s age: METs=4.7+(0.97×VSAQ score)−(0.06×age).

Furthermore, to interpret the clinical relevance of these continuous changes, we explicitly define the minimal clinically important difference as an absolute increase of ≥0.5 METs from baseline [16]. Achieving this prespecified threshold at any point during the follow-up will be interpreted as a clinically meaningful improvement in the patient’s exercise tolerance.

### Secondary Outcome Measures

The secondary outcome measures are as follows:

- 6MWT: Used to objectively evaluate the patient’s exercise tolerance and physical activity level, complementing the VSAQ.
- KCCQ: Used to comprehensively assess changes in the patient’s symptom burden, functional limitations, and overall quality of life.
- NYHA functional class and echocardiographic parameters: Used to evaluate improvements in macroscopic cardiac pump function and structural cardiac abnormalities.
- NT-proBNP: Used to objectively reflect the degree of neurohormonal activation and the disease burden of HF.

### Exploratory Outcome Measures

The TCM syndrome score was chosen as an exploratory outcome measure in this study because of the clinical feature of “syndrome differentiation and treatment” that is stressed in TCM. The objective was to track the improvement trends of particular TCM syndrome clusters after exposure to YQFM. To investigate long-term prognostic patterns and support the overall safety evaluation, clinical end-point events that were tracked over the course of the 90-day follow-up period will also be combined and examined as an exploratory auxiliary outcome.

This study creates a thorough evaluation system that links objective exercise capability, subjective quality of life, heart anatomical and biochemical data, and distinctive TCM symptoms by methodically integrating the aforementioned measurements. This strategy complements the current therapeutic trend that emphasizes holistic therapy in cardiovascular disorders and allows for a more thorough depiction of the practical advantages experienced by patients with HF (Textbox 2).

Textbox 2. **Outcomes.**

**Primary outcome**

- Veterans Specific Activity Questionnaire

**Secondary outcomes**

- Kansas City Cardiomyopathy Patient Quality of Life Scale
- New York Heart Association cardiac function classification
- 6-Minute walk test
- Echocardiogram
- N-terminal pro-B-type natriuretic peptide

**Exploratory outcomes**

- Chinese Medicine Symptom Score
- Clinical end-point events

### Clinical End-Point Events and Safety Evaluation

This study will rigorously monitor and document clinical end-point events and adverse reactions in individuals over a 90-day period in order to undertake a thorough evaluation of safety and long-term benefits.

### Definition and Analysis of Clinical End-Point Events

All-cause mortality, cardiovascular events (cardiovascular death, nonfatal myocardial infarction, or nonfatal stroke), and HF-related events (readmission for worsening HF or the need for intensified treatment with intravenous medications) are all included in the clinical end-point events defined in this study. To address competing risks and bias, these events play a dual role in the analysis: when managing missing data in the LMM analysis for continuous outcomes like the VSAQ, they first act as the basis for informative censoring. Second, they act as an exploratory auxiliary end point; although the sample size was not specifically powered for hard end points, the between-group incidence rates of these events will still be compared to explore trends in long-term benefits and to supplement the safety evaluation.

### Safety Monitoring and Adverse Event Reporting

Given that this study involves intravenous intervention with YQFM, the research team has developed a safety monitoring plan specifically tailored for injection therapy. During the 10-day exposure period, health care professionals will strictly control the infusion rate in accordance with the package insert, focusing on the monitoring of acute infusion reactions and severe allergic reactions during and within 24 hours after infusion. Throughout the entire follow-up period, the onset time, severity, medical interventions undertaken, and outcomes of all adverse events and SAEs will be faithfully recorded.

The National Center for Adverse Drug Reaction Monitoring’s 5-level causation evaluation criteria—certain, probable or likely, possible, improbable, and unrelated—will be used in this study’s attribution standards for safety occurrences. The temporal plausibility of drug administration, dechallenge response, and rechallenge response will be used to independently determine the causal association with the drugs (YQFM or GDMT). The principal investigator and the institutional review board must be informed of any SAE within 24 hours of becoming aware of it.

### Data Management and Quality Control

#### Data Collection and Management

To guarantee privacy, patient identity information was numerically coded. Two separate operators double-entered all of the original clinical data into the electronic data capture system after it had initially been captured on paper case report forms. Data managers regularly evaluated the numerical logic of the case report form indicators, and the system automatically tested for logical flaws and dubious parameters throughout the data entry process. Until the data were entirely accurate, the clinical research associate used data clarification forms to notify the investigators of any missing or erroneous data. The database was locked and then exported for statistical analysis after a blinded examination verified that it was error-free.

#### Bias Minimization and Blinded Assessment

##### Blinded Assessment of Objective Outcomes

All 6MWTs and echocardiographic examinations were measured and interpreted by dedicated technicians and echocardiographers who were separate from the core research team and completely unaware of the patients’ group allocation and medication regimens.

##### Standardized Questionnaire Administration

To reduce expectation bias, literate patients used self-administered questionnaires for subjective markers such as the VSAQ, KCCQ, and TCM syndrome scores. An impartial research assistant blinded to group allocation performed neutral interviews with patients who had visual impairments or writing problems using standardized instructions.

### Standardization, Validity, and Reliability of TCM Scale

The Guiding Principles for Clinical Research on New Traditional Chinese Medicines in China guided the development of the Qi and Yin deficit syndrome score scale for CHF used in this study. As a widely recognized standardized clinical assessment tool in the field of cardiovascular TCM in China, this scale is based on high-level expert consensus, possesses good content validity, and has been extensively applied in numerous previous TCM intervention trials for HF. To maximize the control of subjective bias, strict standard operating procedures were implemented: all evaluating physicians were required to complete unified training and standardized practice sessions. Two independent TCM practitioners performed the formal assessments in a double-blind, “back-to-back” fashion, with a third-party expert resolving any disagreements. During the data verification phase, a subset of samples was randomly selected to calculate interrater reliability. A Cohen κ coefficient of ≥0.75 was set as the target standard for the quality control of the scale.

### Adherence Monitoring

The study monitored medication adherence through a combination of “medication reconciliation+patient medication diaries+investigator interviews during visits.” For patients, medication boxes were collected, and the actual amount of medication consumed was verified at the last follow-up visit. Instances of consecutive missed doses, premature discontinuation, or treatment interruption followed by resumption were all recorded according to the actual exposure status.

### Criteria for Loss to Follow-Up

During the follow-up phase, if a participant did not complete an on-site visit within the specified time frame, the research team prioritized tracing by phone, WeChat, or contact with family members to augment key end-point data. A patient was legally deemed lost to follow-up if they could not be located after 3 consecutive phone follow-up attempts at separate times and if their latest survival status or clinical event outcomes could not be determined despite extensive tracing efforts. The particular reasons for loss to follow-up (eg, withdrawal of informed consent, loss of contact, and relocation) were thoroughly documented and presented in the final findings report using a patient flow diagram.

### Statistical Analysis

Statistical analyses were performed using SPSS (version 29.0; IBM Corp) and R (version 4.4.2; R Foundation for Statistical Computing). Continuous variables with normal or nonnormal distributions were expressed as mean (SD) or median (IQR), respectively. Categorical variables were presented as frequencies (percentages). To control for baseline confounding inherent in the nonrandomized design, the causal estimand was defined as the average treatment effect on the treated, and propensity score inverse probability of treatment weighting (IPTW) was used to balance intergroup differences. The propensity model incorporated variables including demographics, disease duration, NYHA functional class, HF phenotype, baseline VSAQ or KCCQ scores, NT-proBNP levels, and GDMT medications. A standardized mean difference of <0.1 was considered to indicate satisfactory baseline balance. In the IPTW-weighted cohort, an LMM was used to evaluate the intervention effect on repeated-measure continuous end points (VSAQ, KCCQ, and 6-minute walk distance). The model included group, time, and their interaction (group×time) as fixed effects, with the individual patient treated as a random effect. The primary assessment point was prespecified at 90 days after exposure, and the Bonferroni correction was applied for multiple comparisons across time points. For ordinal outcomes such as NYHA functional class, generalized estimating equations or ordered logistic regression were used. Prespecified subgroup and interaction analyses (phenotype×intervention) were conducted based on HF phenotypes to evaluate treatment effect heterogeneity. Model diagnostics for LMM and generalized estimating equations were performed via residual analysis. Safety data were analyzed using descriptive statistics. Missing data were handled using multiple imputation under the missing-at-random assumption. Finally, sensitivity analyses were conducted using the per-protocol set and Rosenbaum’s bounds method (to assess the impact of unmeasured confounding). All statistical inferences were based on 2-sided tests with a significance level of α=.05.

### Ethical Considerations

This study followed the Declaration of Helsinki and the appropriate standards governing clinical research in China. The Medical Ethics Committee of the First Teaching Hospital of Tianjin University of Traditional Chinese Medicine accepted the study protocol (TYLL2024[K]026). The scheme and its informed consent form met the ethical and scientific requirements and have been registered in the International Traditional Medicine Clinical Trial Registry Platform (ITMCTR2024000541). Prior to recruitment, the investigators thoroughly informed the participants of the study’s aims, methods, potential dangers, and the ability to withdraw at any time. Prior to participation, all individuals freely signed a written informed consent form. To maintain participant privacy, all participant identity information was numerically coded, and both electronic data and original paper records were securely stored with restricted access. Participants did not receive any financial compensation for their involvement in this study.

## Results

This work was funded by a research grant from the Tianjin Municipal Health Commission in December 2023 (2024002). Patient recruitment officially began in June 2024 at the First Teaching Hospital of Tianjin University of Traditional Chinese Medicine and Qian’an Hospital of Traditional Chinese Medicine, strictly following the medical ethics committee’s approval granted on May 29, 2024. Although the clinical trial registration process was initiated concurrently with the start of recruitment, the formal public registration on the International Traditional Medicine Clinical Trial Registry Platform (ITMCTR2024000541) was finalized on October 12, 2024, due to the administrative and technical review procedures of the registry platform. We explicitly confirm that this registry record faithfully reflects the original, unamended study protocol. No clinical outcome data were accessed, examined, or analyzed by the research team prior to the completion of this registration. Enrollment and follow-up for all patients were completed by December 2025. Data cleaning and statistical analysis began in January 2026, with the full research findings scheduled to be released in the fall of 2026.

## Discussion

### Anticipated Principal Findings

This prospective, observational cohort research is to assess the real-world efficacy of YQFM as an adjuvant to GDMT on exercise tolerance in patients with CHF and Qi and Yin-deficient syndrome. Our anticipated main findings are that, when compared to patients receiving standardized GDMT alone, adding a 10-day YQFM exposure significantly improves exercise tolerance at 90 days after exposure (as evidenced by significant increases in METs assessed by the VSAQ and the 6-minute walk distance). Furthermore, it is projected to significantly enhance patients’ quality of life (elevated KCCQ scores) and heart function (better NYHA functional class and lower NT-proBNP levels) while lowering the risk of major adverse events. These results will provide strong evidence-based support for the use of TCM as an adjunct in cardiac rehabilitation by directly addressing the increasingly significant core clinical challenges of “decreased exercise tolerance and impaired quality of life” in patients with CHF.

### Comparison to Prior Work

CHF is the last stage of several cardiovascular disorders. The prevalence of HF has been growing annually due to longer survival rates for patients with HF, an aging population, rising prevalence of chronic diseases such as coronary atherosclerotic heart disease, hypertension, diabetes, and obesity, and overall developments in medical treatment [17]. Although long-term, standardized GDMT is the foundation of CHF treatment, data from the Change the Management of Patients with Heart Failure registry show that clinical target attainment rates are alarmingly low: only 10% to 25% of patients achieve the recommended doses for individual GDMT medications, and only 1% achieve the target doses for all GDMT drugs at once. Additionally, patients often have problems such as angioedema and diuretic resistance [18,19]. Most importantly, there are still no effective treatment approaches to cope with the substantial quality of life damage that is mostly caused by reduced exercise tolerance.

The traditional prescription Shengmai San, which is recorded in Yixue Qiyuan, is the source of YQFM. It combines the proven clinical effectiveness of Shengmai San with the quick start of action of an injectable formulation. Research has shown that YQFM improves mitochondrial function by regulating reactive oxygen species generation and the calmodulin-dependent protein kinase II signaling pathway; inhibits the release of different inflammatory mediators like tumor necrosis factor-α, nuclear factor-kappa B, interleukin-6, and interleukin-1β; and prevents apoptosis by regulating phosphoinositide 3-kinase/protein kinase B and adenosine monophosphate–activated protein kinase activation as well as inhibiting mitogen-activated protein kinase [20-22]. According to earlier meta-analyses, YQFM can enhance LVEF and the 6-minute walk distance in patients with HF while lowering BNP and NT-proBNP levels [23,24]. Recent re-evaluations of these systematic reviews, however, have shown that prior data were often of low quality due to methodological flaws such as unclear allocation concealment and a lack of rigorous sample size estimate. More significantly, most earlier research has used HF readmission rates or all-cause mortality as their main outcomes, generally ignoring the crucial factor of exercise tolerance [25].

Grounded in real-world clinical practice, this study innovatively designates “exercise tolerance” as the core outcome measure. The study design references findings from the China Heart Failure Registry, which indicate that the average length of hospital stay for patients with HF is approximately 10 days. Combined with previous research demonstrating that a 7- to 10-day treatment course yields statistically significant clinical benefits, a 10-day exposure period was established for this trial [26-28]. Maintaining adequate GDMT for as long as possible is crucial for the management of patients with CHF. Setting the final follow-up at 90 days profoundly aligns with the critical concept of the “vulnerable phase” in modern HF management. The vulnerable period is commonly defined as the first 1 to 3 months after hospital release or an acute exacerbation of HF. During this time, patients exhibit significant hemodynamic instability and strong neurohormonal activation, which increases the risk of readmission and cardiovascular death [29,30]. As a result, this study undertakes thorough follow-ups at 30, 60, and 90 days after YQFM exposure, intending to cover the full high-risk vulnerable phase [31]. This design not only dynamically evaluates the medium- to long-term intervention effects of YQFM on improving exercise tolerance but also hopes to verify whether this intervention can assist patients in safely navigating this critical window, breaking the vicious cycle of “discharge-worsening-readmission.”

Although this study, using a prospective observational cohort design, is dedicated to validating the real-world clinical efficacy of YQFM combined with GDMT in improving exercise tolerance in patients with CHF, an inevitable question of profound pathophysiological significance arises: given the generally short pharmacokinetic half-life characteristic of TCM injections, how can a brief, 10-day intensive intravenous exposure to YQFM sustain clinically significant efficacy throughout a 90-day follow-up period after drug discontinuation? Its profound biological core may highly depend on the epigenetic reprogramming and multimechanistic cascade regulatory networks triggered by the multicomponent, multitarget interventions of TCM. At the epigenetic level, the progressive deterioration of HF is essentially the consolidation of pathological transcriptional programs in cardiomyocytes, a process closely associated with chromatin remodeling imbalances and dysregulated noncoding RNA expression profiles [32]. Substantial prior fundamental molecular pharmacology research has demonstrated that YQFM and its key bioactive monomers (eg, ginsenoside Rb1) act as potent epigenetic modulators. They intervene in the catalytic activity of histone deacetylases and significantly reverse pathological microRNAs closely linked to cardiac hypertrophy and interstitial fibrosis (eg, targeted inhibition of pro-fibrotic miR-21 alongside the upregulation of cardioprotective miR-542‐3p), thereby effectively erasing the pathological alterations of ventricular remodeling at the transcriptional level [33-35]. This benign epigenetic reprogramming landscape, established during the critical window of the HF vulnerable phase, can sustainably maintain a protective transcriptomic homeostasis in the myocardium via cellular-level metabolic memory, even after the active drug molecules have been completely cleared from the systemic circulation. Furthermore, in the dimension of pleiotropic intervention, early intervention with YQFM during the acute hospitalization phase (ie, the first 10 days) fundamentally curtails the 2 core pathological hubs driving HF progression: neurohumoral overactivation and mitochondrial energy metabolism exhaustion. On the one hand, YQFM effectively buffers the persistent cardiotoxicity induced by the extreme hyperactivity of the sympathetic nervous system and the renin-angiotensin-aldosterone system by antagonizing stress signaling pathways such as mitogen-activated protein kinase and Janus kinase 2/signal transducer and activator of transcription 3 [36,37]. On the other hand, YQFM robustly upregulates peroxisome proliferator-activated receptor-γ coactivator-1α and its downstream metabolic transcriptional networks, massively driving the biogenesis of structurally intact and functionally sound nascent mitochondria within cardiomyocytes [38]. This not only rectifies energy metabolic reprogramming during the progression of HF but also fundamentally halts reactive oxygen species burst-dependent apoptotic cascades. Such impacts on the local cardiac microenvironment, systemic inflammatory networks, and energy substrate metabolism during the decompensated phase completely shatter the pathological vicious cycle that precipitates ventricular remodeling. In summary, short-term intensive exposure to YQFM not only macroscopically improves the heart’s intrinsic contractile function and structural remodeling but also broadly optimizes peripheral skeletal muscle microcirculation and systemic energy substrate allocation. The long-term extension of this series of microscopic mechanisms establishes a robust pathophysiological and theoretical foundation for the sustained, half-life−transcending improvements in exercise tolerance and quality of life observed in patients during the mid-to-late follow-up periods.

This study develops a multifaceted, pragmatic, and scientific evaluation approach for the choice of outcome measures. Decreased exercise tolerance (typically manifesting as reduced skeletal muscle activity due to fatigue or dyspnea) is the core reason for the decline in quality of life among patients with HF [39]. Despite being the gold standard for assessing exercise tolerance, the peak oxygen consumption (VO_2_PEAK_) measured by the symptom-limited cardiopulmonary exercise test is costly, time-consuming, and prone to causing cardiac events and musculoskeletal injuries in older patients [40]. As a result, finding an alternate test that is simple to administer, safe, and appropriate for all participants is critical. The primary end point of this trial was the VSAQ, a safe and simple tool for predicting METs. The VSAQ is a list of everyday activities organized in a progressive order of 13 METs, including dressing, eating, housework, outdoor activities, sports, and others [41]. In an exercise testing investigation including 337 male patients, the VSAQ was shown to be the best predictor reflecting age-related changes in cardiopulmonary fitness, and it has also been shown to reliably predict MET levels and treadmill-measured VO_2_PEAK_ [42,43].

The 6MWT (which shares predictive value with the cardiopulmonary exercise test), the KCCQ (strongly correlated with hospitalization and mortality outcomes in patients with HF), the NYHA functional class, and BNP or NT-proBNP levels are added to the study to assess exercise tolerance multidimensionally. When combined, these provide a thorough evaluation chain that includes biomarkers, subjective quality of life, and objective exercise ability.

### Strengths and Limitations

Both methodological and clinical translational strengths are present in this investigation. To better reflect actual clinical situations, the study first uses a prospective, nonrandomized observational cohort design that is patient-centered and guideline-directed. This real-world prospective design respects patient preferences and more accurately captures the actual treatment choices and medication adherence of complex patients with HF in routine clinical management than RCTs with stringent inclusion and exclusion criteria and highly homogeneous patients. Strict adjustment techniques, such as IPTW based on propensity scores, were prespecified in the statistical analysis plan to mitigate the possible bias brought about by nonrandomized allocation. This approach aims to provide prospective evidence for causal inference with high internal validity while remaining grounded in the context of clinical decision-making, thereby addressing the limited external generalizability typical of traditional RCTs. Second, by choosing exercise tolerance as the main outcome, this study closes a significant gap left by earlier studies that primarily concentrated on hard end points like readmission or all-cause mortality, directly addressing the fundamental clinical issue that significantly affects the quality of life for patients with HF. Finally, the study combines objective measures such as the KCCQ, the 6MWT, echocardiography, and TCM syndrome scores with the VSAQ to estimate METs. This makes it possible to assess the clinical effectiveness of integrating Western medicine and TCM in a thorough and integrated manner.

However, there are certain restrictions on this study. First and foremost, rather than using randomization, patients were assigned to the YQFM and non-YQFM groups based on their preferences and the actual clinical care they received while in the hospital. This suggests that there could be fundamental, substantial disparities between the 2 groups at baseline. As an observational study, it is still challenging to completely eliminate selection bias and unmeasured residual confounding, even though strict adjustment techniques like IPTW were prespecified in the statistical analysis plan to maximally balance differences in known baseline covariates between the groups. This is especially important for subjective outcome measures that rely heavily on patient self-evaluation, like the VSAQ and KCCQ, because patient preference-based allocation is very vulnerable to anticipation bias and the placebo effect. Therefore, the differences in exercise tolerance observed subsequently cannot completely rule out the influence of minor baseline discrepancies. Consequently, we must remain objective and cautious when interpreting this observational evidence for causal inference regarding the independent intervention effect of YQFM. Second, the total sample size of this study may be insufficient to detect smaller differences in effect sizes, and it may specifically lack adequate statistical power in subgroup analyses. Furthermore, the study is geographically restricted to medical institutions in specific regions, and the regional nature of the sample may limit the external generalizability of the findings.

### Future Directions and Dissemination Plan

Building on the exploratory findings and admitting the study’s limitations, additional multicenter, large-scale, randomized, double-blind, controlled studies are needed to confirm the long-term effectiveness and safety of this intervention. In terms of dissemination, while strictly protecting patient privacy, the research team will compile the comprehensive clinical data and analytical results into a high-quality academic paper for submission to an internationally influential, open-access, peer-reviewed cardiovascular journal. We hope that the real-world evidence gathered from this study will give strong evidence-based support for optimizing TCM’s and Western medicine’s combined preventive, therapy, and cardiac rehabilitation methods for CHF.

### Conclusions

This study uses a rigorous, patient-centered, and guideline-directed design to assess the therapeutic effectiveness of YQFM in increasing exercise tolerance in patients with CHF. To appropriately assess the intervention’s effects, we used outcome measures that have demonstrated therapeutic value. The ultimate goal of this study is to increase the level of evidence for YQFM in the cardiac rehabilitation of CHF, optimize the prevention, treatment, and rehabilitation systems for HF, and provide high-quality, evidence-based support for the use of TCM in the management of CHF.

## Abbreviations

6MWT: 6-minute walk test
BNP: B-type natriuretic peptide
CHF: chronic heart failure
GDMT: guideline-directed medical therapy
HF: heart failure
IPTW: inverse probability of treatment weighting
KCCQ: Kansas City Cardiomyopathy Questionnaire
LMM: linear mixed-effects model
LVEF: left ventricular ejection fraction
MET: metabolic equivalent
NT-proBNP: N-terminal pro-B-type natriuretic peptide
NYHA: New York Heart Association
RCT: randomized controlled trial
SAE: serious adverse event
TCM: traditional Chinese medicine
VSAQ: Veterans Specific Activity Questionnaire
YQFM: Yiqi Fumai lyophilized injection
